# Supplementary material for: The interactome of intact mitochondria by cross-linking mass spectrometry provides evidence for coexisting respiratory supercomplexes
Source: Mol Cell Proteomics. 2017 Dec 8;17(2):216–32. doi: 10.1074/mcp.RA117.000470 (PMC5795388; doi:10.1074/mcp.RA117.000470)
Supplement: Supplemental Data [file supp_RA117.000470_133922_0_supp_23978_fzffwf.zip › spectra_annotation/mito_spectra_annotation/147-1-4-1-3-1.pdf]

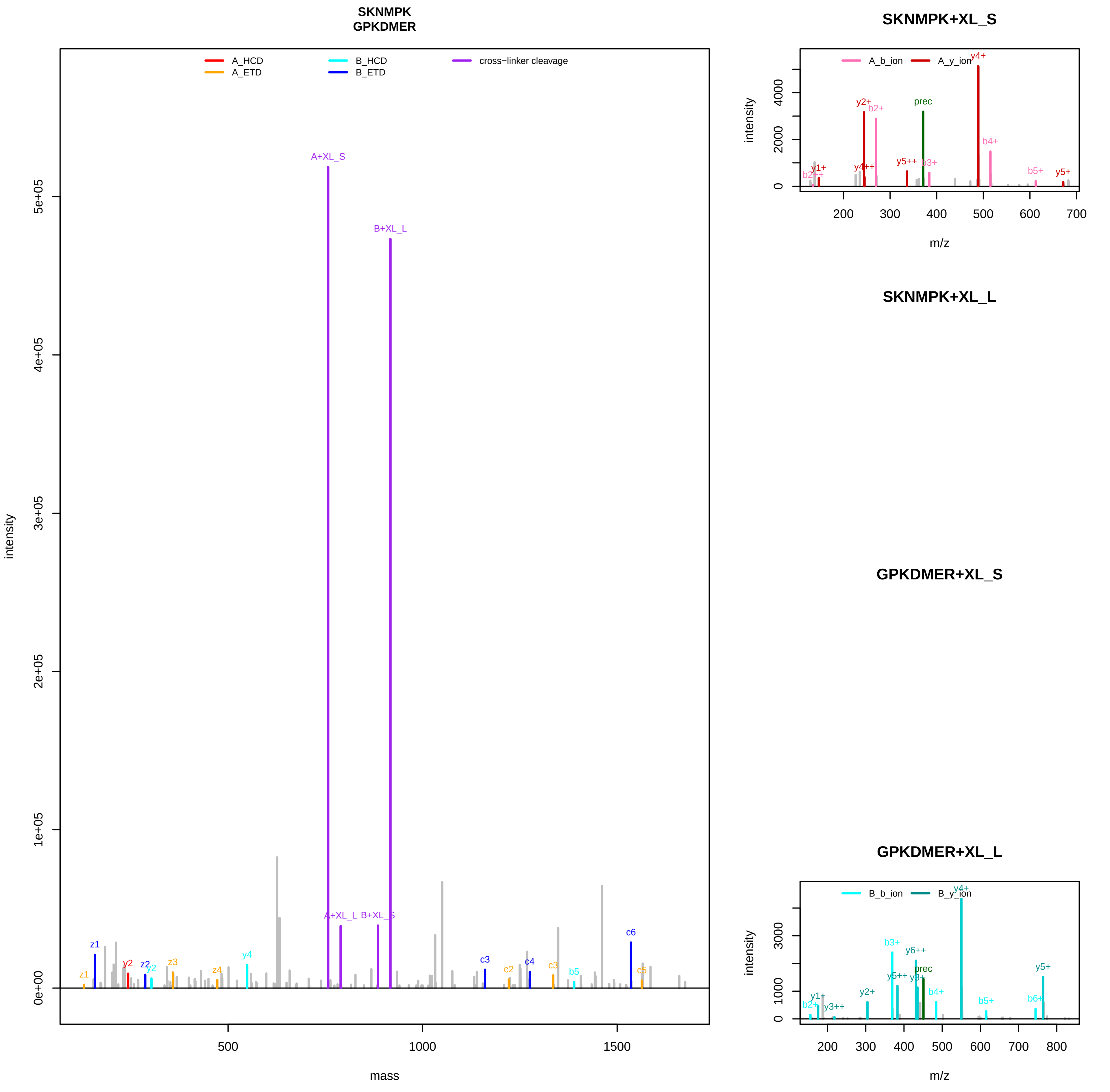
SKNMPK
GPKDMER
A_HCD
A_ETD
B_HCD
B_ETD
cross−linker cleavage
A+XL_S
B+XL_L
A+XL_L
B+XL_S
z1
y2
z2
y2
z3
z4
y4
c3
c2
c4
c3
b5
c6
c5
intensity
mass
0e+00
1e+05
2e+05
3e+05
4e+05
5e+05
500
1000
1500
SKNMPK+XL_S
A_b_ion
A_y_ion
y4+
y2+
b2+
prec
b4+
y5++
b3+
y4++
y1+
b2++
b5+
y5+
intensity
m/z
0
2000
4000
200
300
400
500
600
700
SKNMPK+XL_L
GPKDMER+XL_S
GPKDMER+XL_L
B_b_ion
B_y_ion
y4+
b3+
y6++
prec
y5++
y3+
y5+
y2+
b4+
y1+
y3++
b2+
b5+
b6+
0
1000
2000
3000
200
300
400
500
600
700
800
